# CD163^+^ macrophages infiltration correlates with the immunosuppressive cytokine interleukin 10 expression in tongue leukoplakia

**DOI:** 10.1002/cre2.228

**Published:** 2019-08-01

**Authors:** Manabu Shigeoka, Yu‐ichiro Koma, Mari Nishio, Takahide Komori, Hiroshi Yokozaki

**Affiliations:** ^1^ Division of Pathology, Department of Pathology Kobe University Graduate School of Medicine Kobe Japan; ^2^ Division of Oral and Maxillofacial Surgery, Department of Surgery Related Kobe University Graduate School of Medicine Kobe Japan

**Keywords:** interleukin‐10, macrophage, tongue leukoplakia

## Abstract

**Objective:**

Accumulating evidence suggests that macrophages are involved in the immunoediting of oral squamous cell carcinoma but the role of macrophages in oral carcinogenesis is unclear. We aimed to clarify the role of macrophages in oral leukoplakia, which is the most common oral potentially malignant disorder from immunotolerance viewpoint.

**Materials and methods:**

The study included 24 patients who underwent surgical resection for tongue leukoplakia. The relationships between macrophage markers and clinicopathological factors were assessed. Conditioned medium was harvested from the CD163^+^ human monocytic leukaemia cell line, THP‐1. The phenotypic alteration of human oral keratinocytes by the conditioned medium treatment was assessed using quantitative reverse transcription‐polymerase chain reaction and enzyme‐linked immunosorbent assay. Moreover, the clinical samples were evaluated using immunohistochemistry.

**Results:**

Tongue leukoplakia tissues with high CD163^+^ macrophage infiltration were associated with significantly higher degrees of epithelial dysplasia, abnormal Ki‐67 expression and cytokeratin13 loss when compared with the tissues with low CD163^+^ macrophage infiltration. In vitro, CD163^+^ THP‐1 conditioned medium induced immunosuppressive molecules, especially interleukin‐10 (IL‐10) in human oral keratinocytes. The IL‐10 expression levels showed significant positive correlations with not only the numbers of FOXP3^+^ regulatory T cells but also that of CD163^+^ macrophages.

**Conclusions:**

In tongue leukoplakia, CD163^+^ macrophages infiltration correlates with immunosuppressive cytokine IL‐10 expression.

## 1 INTRODUCTION

The oral cavity plays an essential role in eating, speaking, swallowing, and facial aesthetics (Matsuhira et al., 2015). Oral squamous cell carcinoma (OSCC) is a solid tumour of epithelial origin that has been reported to affect approximately 400,000 people annually worldwide (Petersen, 2009). The mortality rate of OSCC has remained largely unchanged for the last several decades, with a 5‐year survival rate <50% (Petruzzi, Cherubini, Salum, & de Figueiredo, 2017). The early detection of OSCC is thus very important for high patient quality of life.

It was reported that most of OSCCs pass through a premalignant stage (Liu et al., 2017). Oral leukoplakia (OL) is the most common oral potentially malignant disorder. OL occurs most frequently in the tongue. OL is defined as a white plaque of questionable risk having excluded other diseases or disorders that carry no increased risk for factor (Woo, 2019). OL is only a clinical term, and its presentation including clinical appearance, colour, and surface type can vary (Irani, 2016; Shafer & Waldron, 1961). It has been reported that approximately 3.5% (0.13–34.0%) of OLs develop into OSCC (Warnakulasuriya & Ariyawardana, 2016). Previous reports have suggested the proliferation marker Ki‐67 and the squamous cell‐differentiation markers cytokeratin (CK) 13 and CK17 may be the factors for biological malignancy of oral atypical epithelium (Kitamura et al., 2012; Kövesi & Szende, 2003; Mikami et al., 2015). For example, immunohistochemical Ki‐67 expression have been sporadically confirmed in the second basal layer of normal epithelium, but many Ki‐67^+^ cells are distributed to the basal and/or more superficial layers of oral precancerous lesions. Additionally, CK13 is expressed in normal epithelium but not precancerous lesions, whereas CK17 is expressed in precancerous lesions but not in normal epithelium (Yagyuu et al., 2015). However, the mechanism of OL development has not been completely elucidated to date. Thus, a better understanding of the underlying molecular mechanisms of oral carcinogenesis is necessary.

Macrophages (MΦs) are considered the main cancer stromal cells, and tumour‐associated MΦ (TAM) infiltration has been found to correlate with a poor prognosis in various cancers (Lewis & Pollard, 2006; Mantovani, Sozzani, Locati, Allavena, & Sica, 2002; Takeya & Komohara, 2016). From an oncological viewpoint, MΦs have two different functions, as tumour suppressive cells (M1 MΦs) and tumour support cells (M2 MΦs; Goerdt & Orfanos, 1999; Mills, Kincaid, Alt, Heilman, & Hill, 2000; Sica et al., 2008). M2 MΦs display specific receptors known as haemoglobin scavenger receptor (CD163), macrophage scavenger receptor I (CD204), and mannose receptor (CD206; Yokozaki, Koma, Shigeoka, & Nishio, 2018). We previously demonstrated that the infiltration of a high number of CD204^+^ TAMs was associated with tumour aggressiveness in oesophageal squamous cell carcinoma cases (Shigeoka et al., 2013; Shigeoka et al., 2015). The results of many studies suggested that CD163 is a useful marker for TAMs that contribute to the progression and development of OSCC (Fujii et al., 2012 **;** He et al., 2014 **;** Troiano et al., 2019; Usami et al., 2013; Yamagata et al., 2017). However, the role of MΦs in early oral carcinogenesis has not been elucidated.

The concept of tumour immunosuppression has been reported by many researchers (Dunn, Bruce, Ikeda, Old, & Schreiber, 2002; Schreiber, Old, & Smyth, 2011) and several studies on human cancers including OSCC have indicated that TAMs suppress anti‐tumour immunity (Kubota et al., 2017; Takeya & Komohara, 2016; Wen et al., 2018). The association between immune dysfunction and MΦs in oral precancerous lesion was also recently demonstrated by several studies (Stasikowska‐Kanicka, Wagrowska‐Danilewicz, & Danilewicz, 2018a; Stasikowska‐Kanicka, Wagrowska‐Danilewicz, & Danilewicz, 2018b; Ye, Zhang, Lu, & Zhou, 2016). However, the precise roles of MΦs in the oral carcinogenesis have not been completely elucidated.

On the basis of this background, to clarify the specific roles of MΦs in oral carcinogenesis, we conducted immunohistochemical analyses with surgically resected tongue leukoplakia (TL) samples and in vitro assays with THP‐1 human monocytic leukaemia cells and human oral keratinocytes (HOKs) from the viewpoint of immunotolerance.

## 2 MATERIALS AND METHODS

### 2.1 Tissue samples

A total of 24 cases of surgically resected TL treated at the Department of Oral and Maxillofacial Surgery, Kobe University Hospital, Japan were included. The patients were nine men and 15 women with an age range of 31–88 years and mean age of 63.2 years. None of the patients received adjuvant chemotherapy or radiotherapy before surgery. All resected specimens were fixed in 10% formalin and embedded in paraffin. Informed consent for their materials and data to be used was obtained from all patients, and the study was approved by the Kobe University Institutional Review Board.

### 2.2 Morphological evaluation

Three pathologists (M. S., Y. K. and H. Y.), blinded to the patients' clinical data of the patients, performed the grading of epithelial dysplasia based on the modified squamous intraepithelial neoplasia system (Yagyuu et al., 2015; Yagyuu et al., 2017). Briefly, the degree of dysplasia was divided into high (moderate/severe dysplasia or carcinoma in situ) and low (no/mild dysplasia) grades. In this study, not only the basaloid‐type but also the differentiated‐type carcinoma in situ were classified as high grade.

### 2.3 Immunohistochemical evaluation

We used a modified version of the immunoglobulin enzyme bridge technique with the Linked Streptavidin‐Biotin Kit (DakoCytomation, Glostrup, Denmark) as described elsewhere (Shigeoka et al., 2013). We used specific mouse monoclonal antibodies to CD163 (1:100, #10D6, Novocastra, Newcastle upon Tyne, UK), CD204 (1:50, #SRA‐E5, TransGenic, Kobe, Japan), CD206 (1:50, #D‐1, Santa Cruz Biotechnology, Santa Cruz, CA, USA), Ki‐67 (1:100, #MIB‐1, DakoCytomation, Glostrup, Denmark), CK13 (1:50, #KS‐1A3, Diagnostic Biosytems, Pleasanton, CA, USA), CK17 (1:40, #E3, DakoCytomation, Glostrup, Denmark), and FOXP3 (1:100, #236A/E7, Abcam, Cambridge, MA, USA) and rabbit monoclonal antibody to interleukin (IL)‐10 (1:400, #ab34843, Abcam, Cambridge, MA, USA) for the primary reaction.

After gentle rinsing with 0.05 M Tris‐HCl, the sections were incubated with biotinylated goat anti‐rabbit or anti‐mouse IgG and streptavidin conjugated to horseradish peroxidase (HRP). Chromogenic fixation was performed by immersing the sections in a solution of 3,3′‐diaminobenzidine. Sections were counterstained with Mayer's haematoxylin. The expressions of Ki‐67, CK13, and CK17 were assessed with a modified version of a previous method (Yagyuu et al., 2015). Briefly, the Ki‐67 expression was evaluated as a second basal layer or an unclear or basal layer and/or more superficial layer. The CK13 and CK17 expressions were evaluated as follows: positive, loss, or unclear. MΦs were each counted in subepithelial areas up to 100 μm from the basement membrane. CD163^+^, CD204^+^, and CD206^+^ round cells were counted as MΦs. Three high‐power fields (×400) were randomly selected, and the mean number was calculated (Sato et al., 2005; Yagyuu et al., 2017; Zhang et al., 2003). The median MΦ number in subepithelial areas was used to divide the patients into high and low groups. IL‐10 immunoreactivity of 24 TL tissue samples was divided into high and low immunoreactivity in comparison with that of corresponding normal oral epithelium. Three pathologists (M. S., M. N., and H. Y.) who were blinded to the clinical data performed these evaluations.

### 2.4 Cell cultures

HOKs were purchased from ScienCell Research Laboratories (Carlsbad, CA, USA). HOKs were incubated in oral keratinocyte medium (ScienCell, Carlsbad, CA, USA), with oral keratinocyte growth supplement (ScienCell, Carlsbad, CA, USA) and penicillin/streptomycin solution (ScienCell, Carlsbad, CA, USA). Because HOK is not an immortalised cell, we did not passage HOK more than five times to avoid excessive passage number. The human acute monocytic leukaemia cell line, THP‐1 was purchased from the American Type Culture Collection (Manassas, VA, USA; Tsuchiya et al., 1980). THP‐1 cells were cultured in RPMI1640 (Wako, Osaka, Japan) with 10% foetal bovine serum (Sigma‐Aldrich, St. Louis, MO, USA) and 1% antibiotic‐antimycotic (Invitrogen, Carlsbad, CA, USA). THP‐1 conditioned medium (CM) was prepared as follows: THP‐1 cells (5 × 10^5^ per well) were stimulated with 100 nM 12‐*O*‐tetradecanoylphorbol 13‐acetate (TPA; Cell Signaling, Danvers, MA, USA) for 48 hr, and the medium was then changed to complete oral keratinocyte medium (ScienCell). Before the medium was changed, to remove TPA sufficiently from the well, we aspirated well and washed using PBS, three times in the present study. After 2 days, the supernatant was harvested, centrifuged, and stored in aliquots at −80°C.

### 2.5 Quantitative reverse transcription‐polymerase chain reaction

Total mRNA was extracted from THP‐1 cells with the use of an RNA extraction kit (RNeasy Kit; Qiagen, Hilden, Germany). The quantitative reverse transcription‐polymerase chain reaction (qRT‐PCR) amplifications of *PD‐L1*, *PD‐L2*, *IL‐10*, *TGF‐β*, and *GAPDH* (control gene) were performed using the ABI StepOne Real‐time PCR system (Applied Biosystems, Foster City, CA, USA). The threshold cycle (Ct) values were determined by plotting the observed fluorescence against the cycle number. The Ct values of *PD‐L1*, *PD‐L2*, *IL‐10*, and *TGF‐β* were analysed using the comparative threshold cycle method and were normalised to the value of *GAPDH.* The relative gene expressions were estimated using the following formula: relative expression = 2 −(Ct [*target gene*]−Ct[*GAPDH*]). Primers were designed according to previous reports (Hasita et al., 2010; Hassan, Akram, King, Dockrell, & Cliff, 2015) as follows. *PD‐L1*: 5′‐AAA TGG AAC CTG GCG AAA GC‐3′ (forward) and 5′‐GAT GAG CCC CTC AGG CAT TT‐3′ (reverse); *PD‐L2*: 5′‐GTC TTG GGA GCC AGG CTG AC‐3′ (forward) and 5′‐TGA AAA GTG CAA ATG GCA AGC‐3′ (reverse); *IL‐10*: 5′‐GGT TGC CAA GCC TTG TCT GA‐3′ (forward) and 5′‐AGG GAG TTC ACA TGC GCC T‐3′ (reverse); *TGF‐β*: 5′‐TGA AAA CTG CAA ATG GCA AGC‐3′ (forward) and 5′‐ACG TAG TAC ACG ATG GGC AGC‐3′ (reverse); *GAPDH*: 5′‐GCA CCG TCA AGG CTG AGA AC‐3′ (forward) and 5′‐TGG TGA AGA CGC CAG TGG A‐3′ (reverse).

### 2.6 Western blot analysis

Cells were lysed in a buffer containing 50 mM Tris‐HCl (pH 7.4), 125 mM NaCl, 0.1% Triton X‐100, and 5 mM ethylenediaminetetraacetic acid with a 1% protease inhibitor cocktail (Sigma, St. Louis, MO, USA). We loaded 36 μg of sample in total volume of 20 μl. The resultant lysates were separated on 5–20% SDS‐polyacrylamide gels, transferred to membranes with iBlot Gel Transfer Stack (Invitrogen), and reacted with mouse anti‐CD163 (1:100, #10D6, Novocastra), mouse anti‐CD204 (1:500, #SRA‐E5, TransGenic), goat anti‐CD206 (1:100, #C‐20, Santa Cruz), and rabbit anti‐GAPDH (1:200, #FL‐335, Santa Cruz). After washing, the blots were incubated with HRP‐conjugated donkey anti‐mouse antibodies (1:1000, #NA934OV, Amersham, UK). The blots were then probed with ImmunoStar Reagents (Wako, Osaka, Japan).

### 2.7 Enzyme‐linked immunosorbent assay

Human IL‐10 concentrations were measured by the Quantkine ELISA Human IL‐10 Immunoassay (R&D, MN, USA) according to the manufacturer's instructions. The optical density of each well was read at 450 and 540 nm. The concentration of IL‐10 was calculated using a standard curve and the measured absorbance.

### 2.8 Statistical analysis

We used the *χ* ^2^ test to analyse the relationships between the patient's clinicopathological features and the immunohistochemistry results. Statistical comparisons were performed using the paired *t* test. All in vitro assay was performed three times independently. A *p* value < .05 was considered statistically significant. All statistical analyses were carried out using SPSS Statistics Ver. 21 software (IBM, Chicago, IL, USA).

## 3 RESULTS

### 3.1 Macrophage infiltration was observed in TL

**Figure 1:**
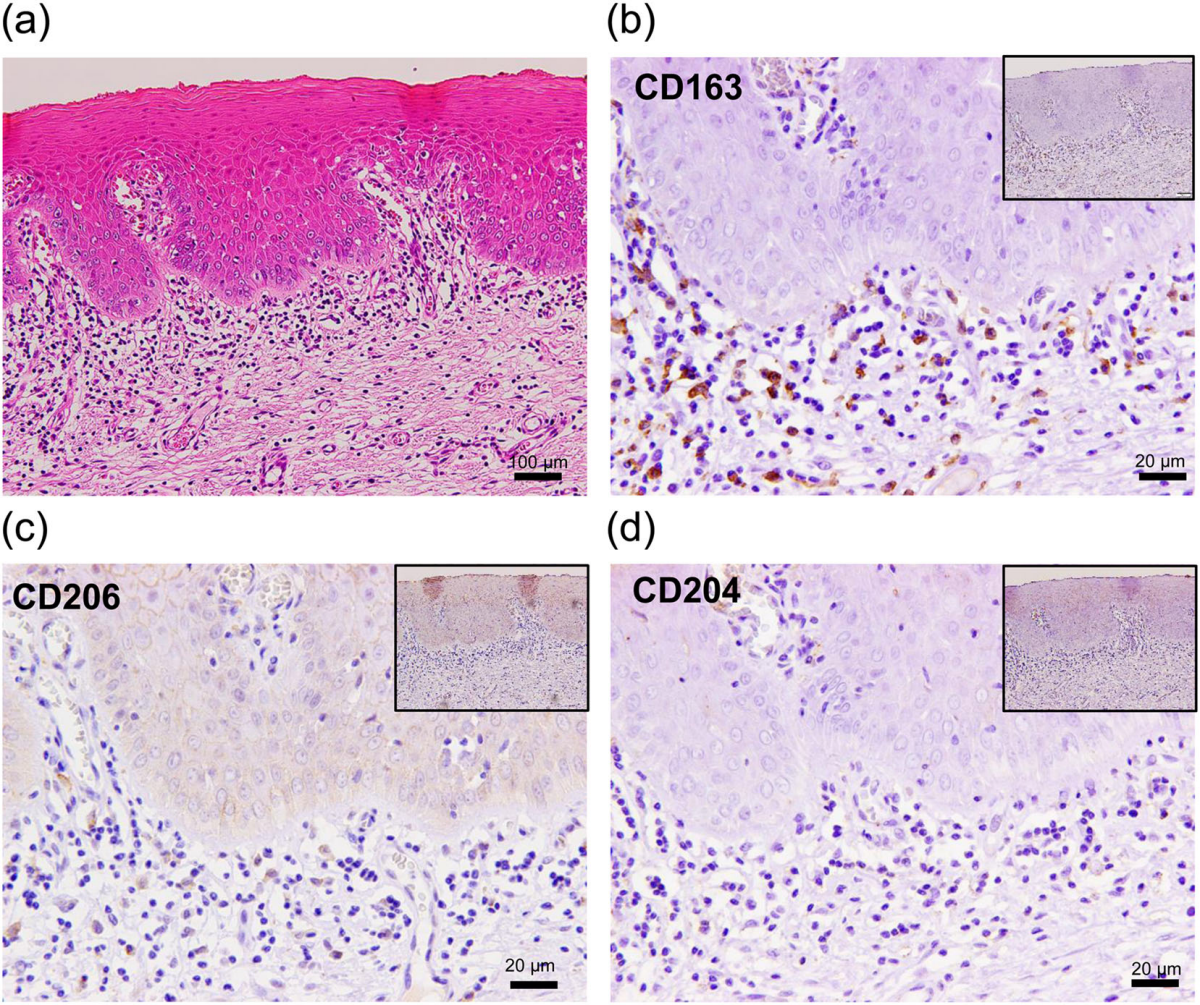
Macrophage infiltration in a representative case of tongue leukoplakia (TL) with moderate or severe dysplasia. (a) A dense infiltration of inflammatory cells was observed in the subepithelial stroma (with original magnification: ×100 and scale bars: 100 μm). (b) Many CD163^+^ cells were distributed beneath the basement membrane. (c) CD206^+^ cells were overtly less in number than CD163^+^ cells. (d) No CD204^+^ cells were observed in the epithelial or subepithelial area (with original magnification: ×400 and scale bars: 20 μm; inset magnification: ×100)

MΦs expressing CD163, CD204, or CD206 immunoreactivity were detected in all TL tissues examined. CD163^+^ MΦs and CD206^+^ MΦs were distributed in the subepithelial stroma, especially beneath the basement membrane, whereas no CD204^+^ MΦs were observed. Moreover, CD206^+^ MΦs were overtly less in number when compared with CD163^+^ MΦs (Figure 1a–d).

### 3.2 The infiltrating number of CD163^+^ MΦs was closely associated with clinicopathological factors of the patients with TL

**Figure 2:**
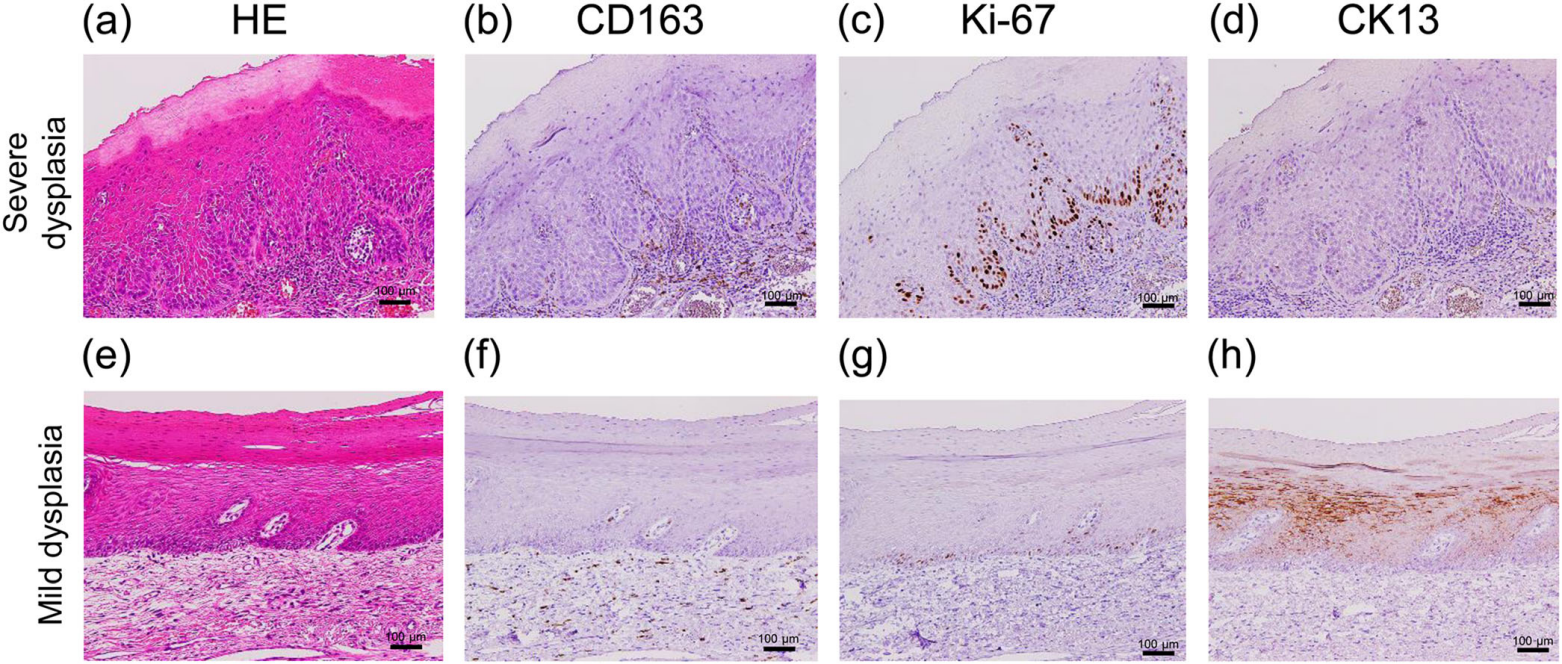
Comparison of immunohistochemical findings between severe dysplasia and mild dysplasia. (a) Representative haematoxylin and eosin (HE) image of tongue leukoplakia (TL) tissue with severe dysplasia. (b) In severe dysplasia, CD163^+^ cells are diffusely observed in the subepithelial area. (c) In severe dysplasia, Ki‐67^+^ cells are distributed in several layers from the basal layer. (d) In severe dysplasia, the expression of CK13 is lost. (e) Representative HE image of TL tissue with mild dysplasia. (f) In mild dysplasia, CD163^+^ cells are occasionally observed in the subepithelial area. (g) In mild dysplasia, Ki‐67^+^ cells are detected in only the second basal layer. (h) In mild dysplasia, CK13 is expressed in epithelial cells (with original magnification: ×100 and scale bars: 100 μm)

We next determined whether CD163^+^ MΦs and CD206^+^ MΦs had any statistical associations with the clinicopathological factors of the patients with TL (Table 1). We divided the TL cases into high‐MΦ and low‐MΦ groups according to the median values of CD163^+^ MΦs (24.5, range 3.3–40.7) and CD206^+^ MΦs (8.0, range 0.3–21.0). A high CD163^+^ MΦ number showed significantly positive associations with the degrees of epithelial dysplasia, abnormal Ki‐67 expression and cytokeratin 13 (CK13) loss in TL tissues (Figure 2a–h). Conversely, a high CD206^+^ MΦ number did not show associations with these clinicopathological factors, except abnormal Ki‐67 expression and the number of non‐drinking patients.

**Table 1:** Infiltration of CD163^+^ cells and CD206^+^ cells in tongue leukoplakia and the associations with clinicopathological parameters

	Number of cases	CD163^+^ cells		*p* value	CD206^+^ cells		*p* value
		Low (*n* = 12)	High (*n* = 12)		Low (*n* = 12)	High (*n* = 12)	
Age:							
Mean	‐	61.9	64.2	.523	61.3	65.0	.456
Median	‐	67	68		67.5	67.5	
Sex:							
Male	9	5	4	.673	6	3	.206
Female	15	7	8		6	9	
Smoking:							
Never	20	11	9	.776	10	10	1.00
Past + present	4	1	3		2	2	
Alcohol intake:							
Never	15	8	7	.673	5	10	.035*
Past + present	9	4	5		7	2	
Clinical appearance:							
Homogenous	18	11	7	.059	11	7	.059
Non‐homogenous	6	1	5		1	5	
Lesion colour:							
White	22	11	11	1.00	12	10	.14
White/red	2	1	1		0	2	
Degree of dysplasia:							
<Mild	14	11	3	.001*	8	6	.408
Moderate or severe	10	1	9		4	6	
Ki‐67							
Second basal layer or unclear	15	10	5	.035*	10	5	.035*
Basal layer and/or more superficial layer	9	2	7		2	7	
CK13							
Positive	5	5	0	.001*	3	2	.407
Loss	15	4	11		6	9	
Unclear	4	3	1		3	1	
CK17							
Positive	5	2	3	.65	1	4	.132
Loss	19	10	9		11	8	
Unclear	0	0	0		0	0	

* *p* value < .05 was considered statistically significant.

### 3.3 CD163^+^ THP‐1 CM induced IL‐10 expression in HOKs

**Figure 3:**
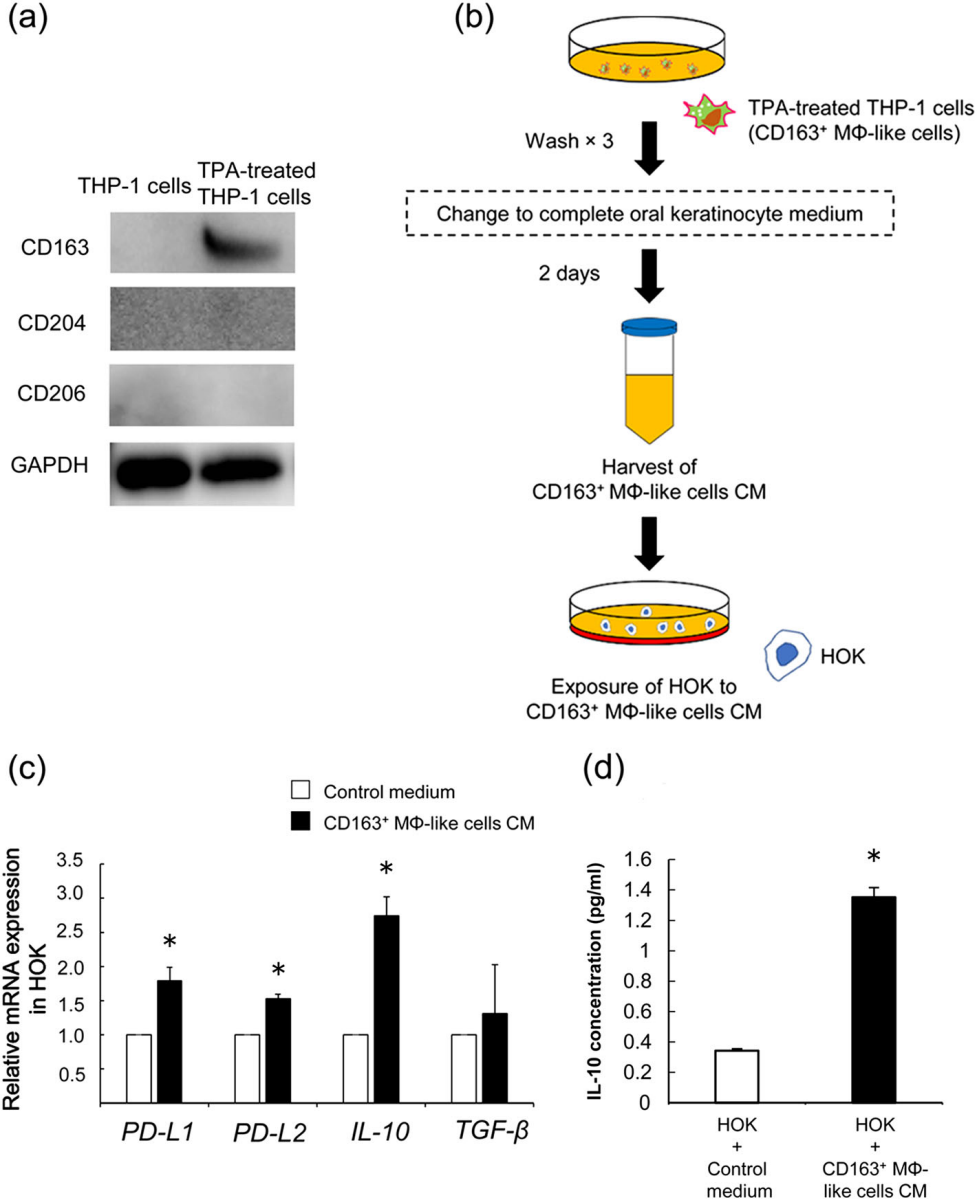
The effects of CD163^+^ MΦ‐like cells conditioned medium (CM) on the human oral keratinocytes (HOKs). (a) Induction of CD163 by TPA (100 nM) in THP‐1 cells is confirmed by the western blot analysis. Data are representative of three independent experiments. (b) Scheme of the treatment of HOKs with TPA‐treated THP‐1 (CD163^+^ MΦ‐like) cells CM. (c) Expressions of immunosuppressive genes induced by CD163^+^ MΦ‐like cells CM. Data are mean ± *SD* of triplicate wells and are representative of three independent experiments (^*^ *p* < .05). (d) IL‐10 secretion from HOKs was significantly induced by CD163^+^ MΦ‐like cells CM. CD163^+^ MΦ‐like cells CM‐treated HOKs or control HOKs cultured in oral keratinocyte medium for 48 hr and the supernatants were analysed by ELISA. Data are mean ± *SD* of triplicate wells and are representative of three independent experiments (^*^ *p* < .05)

On the basis of the immunohistochemical findings, we hypothesised that infiltrating CD163^+^ MΦs in the subepithelial areas of TL tissues contributed to the process of oral squamous cell carcinogenesis. We investigated the role of MΦs in HOKs in vitro. First, to induce MΦ‐like differentiation, 5 × 10^6^ THP‐1 cells were treated with 100 nM TPA for 2 days. Then, in the THP‐1 cells, the expressions of CD163, CD204, and CD206 cytological markers for M2 MΦs used in immunohistochemical analyses of TL tissues were assessed. Significant induction of CD163 but not CD204 and CD206 by TPA treatment was observed on western blot analysis (Figure 3a). As we immunohistochemically confirmed that CD204^+^ MΦs were rarely detected and the number of CD206^+^ MΦs was smaller than that of the CD163^+^ MΦs in the 24 TL tissues. We prepared the supernatant of TPA‐treated THP‐1 cells as CD163^+^ MΦ‐like cells CM (Figure 3b). We investigated the immunosuppressive genes from HOK stimulated with CD163^+^ MΦ‐like cells CM. Interestingly, the results of the qRT‐PCR analysis demonstrated that *PD‐L1, PD‐L2*, and *IL‐10* mRNA expressions in HOKs were significantly induced by CD163^+^ MΦ‐like cells CM, whereas *TGF‐β* expression in HOKs was not significantly altered (Figure 3c). Among these genes, we decided to focus on *IL‐10*, which was the most increased in HOKs by CD163^+^ MΦ‐like cells CM. Moreover, we observed that CD163^+^ MΦ‐like cells CM induced IL‐10 secretion from HOK by ELISA (Figure 3d).

### 3.4 The expression levels of IL‐10 showed a significant positive correlation with not only the numbers of regulatory T cells but also CD163^+^ MΦs and in TL

**Figure 4:**
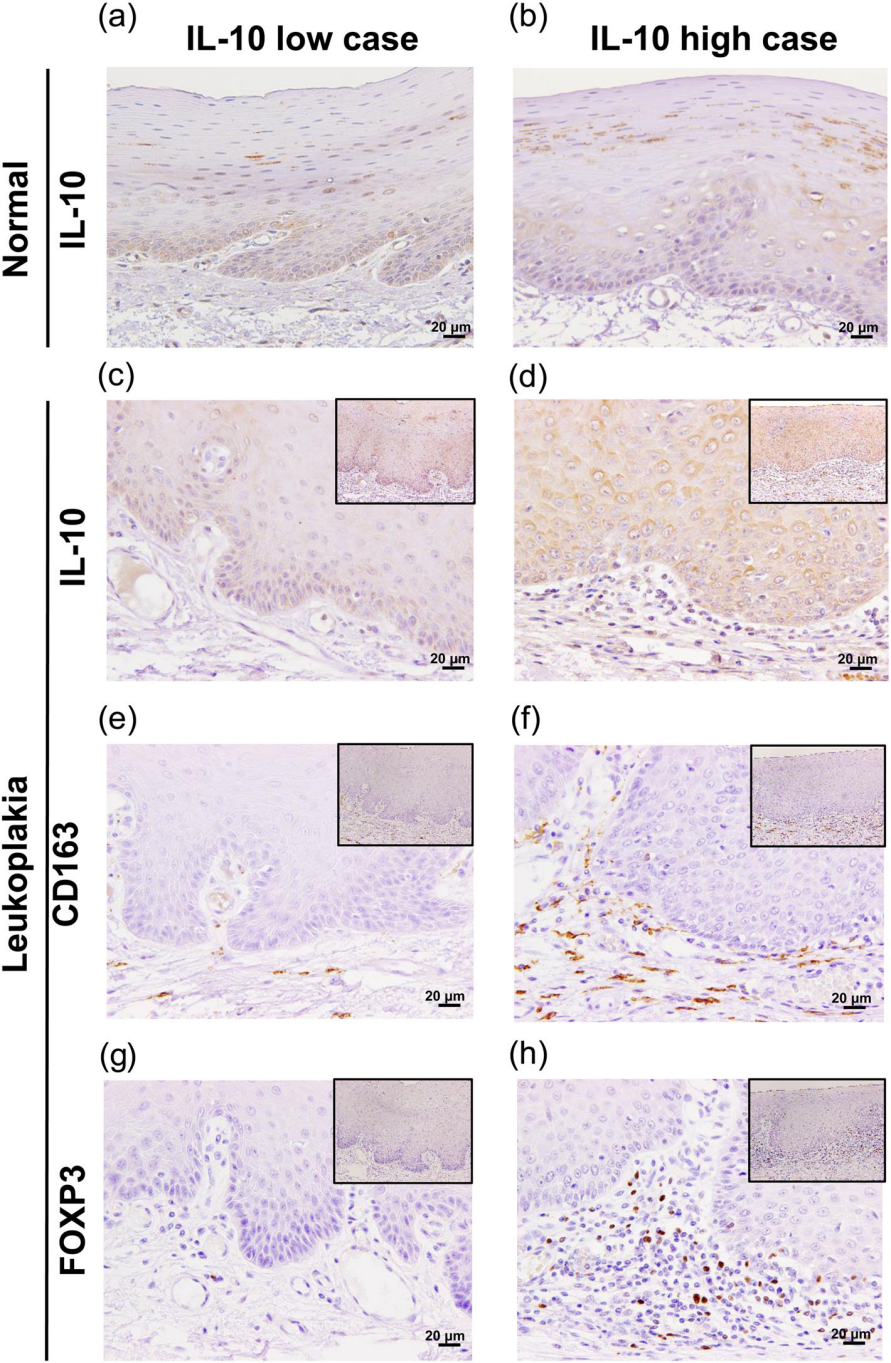
High expression of IL‐10 show a significant positive correlation with the numbers of CD163^+^ MΦs and regulatory T cells in tongue leukoplakia (TL). (a,b) Immunohistochemical images of IL‐10 expressions in normal epithelium of representative TL cases. (c,d) Immunohistochemical images of high and low IL‐10 levels in representative TL cases. (e–h) The expression levels of CD163^+^ cells and FOXP3^+^ cells are stronger in the IL‐10 high group compared with the IL‐10 low group (with original magnification: ×400 and scale bars: 20 μm; inset magnification: ×100)

We next investigated whether the level of IL‐10 expression in the epithelium of the TL tissues had any statistical association with the infiltration of MΦs or regulatory T cells (Tregs). We evaluated the levels of IL‐10 immunoreactivities in the epithelium of the TL tissues using corresponding normal epithelia as a control. We also counted FOXP3^+^ cells as Tregs. The levels of IL‐10 expression showed a significant positive correlation with the infiltrating Tregs. Interestingly, the tissue samples with high IL‐10 expression were significantly correlated with the infiltrating CD163^+^ MΦs (Figure 4a–h; Table 2).

**Table 2:** Expression levels of IL‐10 in tongue leukoplakia and the associations with the infiltration of MΦs and regulatory T cells

	Number of cases	IL‐10		*p* value
		Low (*n* = 14)	High (*n* = 10)	
CD163^+^ cells				
Low	12	10	2	.013*
High	12	4	8	
CD206^+^ cells				
Low	12	7	5	1.00
High	12	7	5	
FOXP3^+^ cells				
Low	12	10	2	.013*
High	12	4	8	

* *p* value < .05 was considered statistically significant.

## 4 DISCUSSION

Several studies have shown that the infiltration of CD163^+^ MΦs positively correlates with epithelial dysplasia and the malignancy of oral precancerous lesion (Mori, Haraguchi, Hiori, Shimada, & Ohmori, 2015; Stasikowska‐Kanicka et al., 2018b). Additionally, previous studies have shown that the number of Tregs and the levels of IL‐10 expression increase in oral precancerous lesions (Goncalves et al., 2017; Sun et al., 2016). However, to the best of our knowledge, this is the first study to demonstrate the association of CD163^+^ MΦs infiltration with IL‐10 expression in TL.

The results of our analyses demonstrated that the number of infiltrating CD163^+^ MΦs but not the numbers of CD206^+^ MΦs and CD204^+^ MΦs had significantly positive correlations with the degrees of epithelial dysplasia, abnormal Ki‐67 expression, and CK13 loss in TL tissues. These results are in agreement with previous findings that CD163^+^ MΦs are the major TAMs in OSCC and that a high number of CD163^+^ MΦs correlates with poor prognosis (Fujii et al., 2012; Wang et al., 2014). It was reported that CD163^+^ MΦs are distributed not only in the cancer stroma but also within the cancer nest in OSCC (Usami et al., 2013). On the other hand, it has been shown that the majority of infiltrating CD163^+^ MΦs were distributed in the subepithelial stroma in OL (Mori et al., 2015). In accordance with these reports, we observed herein that CD163^+^ MΦs were distributed in the subepithelial stroma, especially beneath the basement membrane. Many reports have shown the importance of a direct interaction between MΦs and cancer cells (Komohara et al., 2013; Komohara, Ohnishi, Kuratsu, & Takeya, 2008; Usami et al., 2013). We speculate that MΦs adhere to cancer cells due to the breakdown of the basement membrane during the carcinogenic process, subsequently resulting in the induction of MΦ‐derived protumour functions.

Moreover, we evaluated the expressions of immunosuppressive genes in HOKs stimulated with CD163^+^ MΦ‐like cells CM. In our study, the TPA‐treated human monocytic leukaemia cell line THP‐1 was used as an efficient model for CD163^+^ MΦs in TL. In accordance with previous reports, our preliminary data demonstrated that TPA‐treated THP‐1 cells polarise into the M2 phenotype with IL‐4 (20 ng/ml) treatment and express not only CD163 but also CD204 and CD206, whereas no obvious induction causes the cells to polarise into M1 with lipopolysaccharide (LPS; 10 ng/ml) and interferon‐gamma (IFN‐γ; 20 ng/ml; data not shown; Stewart, Yang, Makowski, & Troester, 2012; Qin, Lai, Landero, & Caruso, 2012; Zhang, Sime, Juhas, & Sjolander, 2013; Gao et al., 2018). In addition, Mori et al. reported that CD163^+^ TAMs in OL co‐express CD163 and STAT1, suggesting that the TAMs in oral premalignant lesions possess an M1 phenotype (Mori et al., 2015). Essa et al. (2016) showed that CD204^+^ cells were clearly fewer in number when compared with CD68^+^/CD163^+^ MΦs in both the subepithelial and intraepithelial zones. Our immunohistochemical analysis of MΦs in TL showed that the number of CD163^+^ MΦs was higher than the number of CD204^+^ MΦs or CD206^+^ MΦs. Considering these findings, we used TPA‐treated THP‐1 cells as the model for MΦs in TL in this study. Further studies are required to determine the MΦ phenotype using bone marrow‐derived peripheral blood monocytes.

We observed that the expressions of immunosuppressive molecules, especially IL‐10 in HOKs were induced by CD163^+^ MΦ‐like cells CM. Moreover, in our immunohistochemical analysis, IL‐10 was detected in epithelial cells of TL tissues.

It is reported that TAMs induce the infiltration and differentiation of Tregs and that Tregs induce MΦ polarisation into the M2 phenotype (Guan et al., 2007). Sun et al. (2016) reported that IL‐10 expression levels gradually increased during the early stages of OL and in OSCC (Sun et al., 2016). Kubota et al. (2017) revealed that TAMs promote T cell regulation via IL‐10 and PD‐L1 production in OSCC (Kubota et al., 2017). Our present findings also showed that the expression level of IL‐10 was significantly positively correlated with the infiltration of Tregs in TL. It is speculated that IL‐10 induced by CD163^+^ MΦs may contribute to the infiltration of Tregs during the development of oral carcinogenesis.

There are three limitations in this study. First, the sample size (*n* = 24) was small. At our hospital, the first choice of treatment for leukoplakia occurring in parts other than the tongue (including the gingiva or the palate) is vaporisation with a CO_2_ laser instead of surgical resection; it is thus difficult to perform several histopathological examinations. Second, we have not verified the effect of IL‐10 on Tregs using in vitro assays. Mori et al. (2015) proposed that the infiltration of MΦs and T cells into epithelial lesions may be involved in early morphological changes in the development of dysplasia (Mori et al., 2015). To overcome these limitations, it may be necessary to undertake an accurate study with a large sample size or an in vitro study with not only HOKs and THP‐1 cells but also with T cells. Third, we did not analyse the nature of the CD163^+^ MΦ‐like cells CM that induced the IL‐10 expression in HOK. We speculated that the humoral factor(s) from CD163^+^ MΦs is one of the important inducers of IL‐10 in TL. It is reported that IL‐10 is produced by monocytes or T cells (Del Prete et al., 1993; Konjevic, Vuletic, Mirjacic Martinovic, Larsen, & Jurisic, 2019). There are many reports on the inducer of IL‐10. The induction of IL‐10 from Tregs is reported to be dependent on TGF‐β, but IL‐10, IL‐2, and IL‐4 also help to promote its optimal production (Josefowicz, Lu, & Rudensky, 2012; Ouyang & O'Garra, 2019). In mouse models of malaria, IL‐27 is an important regulator of IL‐10 producing Type 1 regulatory T (Tr1) cells (Kumar, Ng, & Engwerda, 2019). Kumar et al. (2019) also showed that Type I interferons (IFNs) are also critical regulators of IL‐10 production by Tr1 cells (Kumar et al., 2019). It is showed that the increasing plasma IL‐10 levels observed during exercise are mediated by IL‐6 from contracting muscles (Steensberg, Fischer, Keller, Moller, & Pedersen, 2003). Comprehensive analysis of CD163^+^ THP‐1 CM using cytokine array or cDNA microarray should be further conducted.

Overall, our results indicated that CD163^+^ MΦs might play important roles in the development of TL via immunosuppression. Immunological approaches targeting IL‐10 could be effective for the establishment of novel therapies of OL.

## AUTHOR CONTRIBUTIONS

M. Shigeoka was responsible for the design of the study, the data analysis, and the drafting of the paper. M. Nishio and YI Koma took part in the histological evaluations. T. Komori contributed to the selection of clinical data. H. Yokozaki participated in the histological evaluations, manuscript design, and supervised the study.

## CONFLICT OF INTERESTS

The authors have no conflict of interest.
